# Supplementary material for: Daytime warming has stronger negative effects on soil nematodes than night-time warming
Source: Sci Rep. 2017 Mar 7;7:108. doi: 10.1038/s41598-017-00218-4 (PMC5428061; doi:10.1038/s41598-017-00218-4)
Supplement: Supplementary file 1 — Supplementary Information [file 41598_2017_218_MOESM1_ESM.pdf]

Daytime warming has stronger negative effects on soil nematodes than night-time warming

Xiumin Yan[1, 5 †], Kehong Wang[2, 5 †], Lihong Song[3, 5*], Xuefeng Wang[4, 5], Donghui Wu[5*]

1 School of Geography and Tourism, Guizhou Education University, Guiyang 550018, PR. China

2 College of Resources and Environmental Science, Chongqing University, Chongqing 40044, PR.China

3 College of Agriculture, Guizhou University, Guiyang 550025, PR. China

4 School of Chinese Medicinal Materials, Jilin Agricultural University, Changchun 130117, PR. China

5 Key Laboratory of Wetland Ecology and Environment, Northeast Institute of Geography and Agroecology, Chinese Academy of Sciences, Changchun 130012, PR. China

*Corresponding authors: Lihong Song (lihong_song@qq.com), Donghui Wu (wudonghui@iga.ac.cn)

† These authors contribute equally.

Legends:

Table 1 Statistic results of nematodes under asymmetric warming.

Fig 1 Soil temperature in control, daytime warming, night-time warming and diurnal warming.

Fig 2 Soil moisture in control, daytime warming, night-time warming and diurnal warming.

genus	Trophic group	Density (Mean ± SE)				*p*
		C	DW	NW	24hW	
Acrobeles	BF	**131.7±21.3**	90.6±22.2	98.1±23.2	*39.2±9.4*	0.010
Acrobeloides	BF	269.2±31.2	*135.8±32.7*	**274.7±38.8**	164.7±40.9	0.011
Aphelenchoides	FF	**51.9±12.1**	*25.2±5.9*	31.5±11.5	29.0±12.5	0.257
Aporcelaimium	OP	0.4±0.4	**3.6±1.2**	1.6±1.2	*0.4±0.4*	-
Eucephalobus	BF	60.0±17.7	32.0±13.4	**61.2±12.4**	*23.2±10.3*	0.042
Eudorylaimus	OP	**10.5±4.9**	-	-	4.1±1.7	-
Paraphelenchus	FF	127.2±30.0	74.5±15.2	**183.3±34.3**	*55.3±20.1*	0.014
Paratylenchus	PP	**17.4±15.4**	*2.2±0.8*	-	3.6±1.7	0.192
Pratylenchus	PP	*2.1±1.4*	4.7±2.0	**6.3±6.3**	4.1±4.1	-
Prismatolaimus	BF	3.1±1.1	*1.1±0.5*	**9.0±2.4**	7.4±1.9	0.003
Protorhabditis	BF	**24.1±6.5**	14.1±4.7	12.5±2.8	*9.3±2.7*	0.262
Rotylenchus	PP	**123.9±32.5**	27.7±15.8	28.1±8.4	*3.4±2.2*	0.000

Trophic group (PP, plant feeders; FF, fungal feeders; BF, bacterial feeders; OP, omnivore-carnivores) was according to Bongers and Bongers (1998). Maximum data were showed in bold. Minimum data were showed in italics.

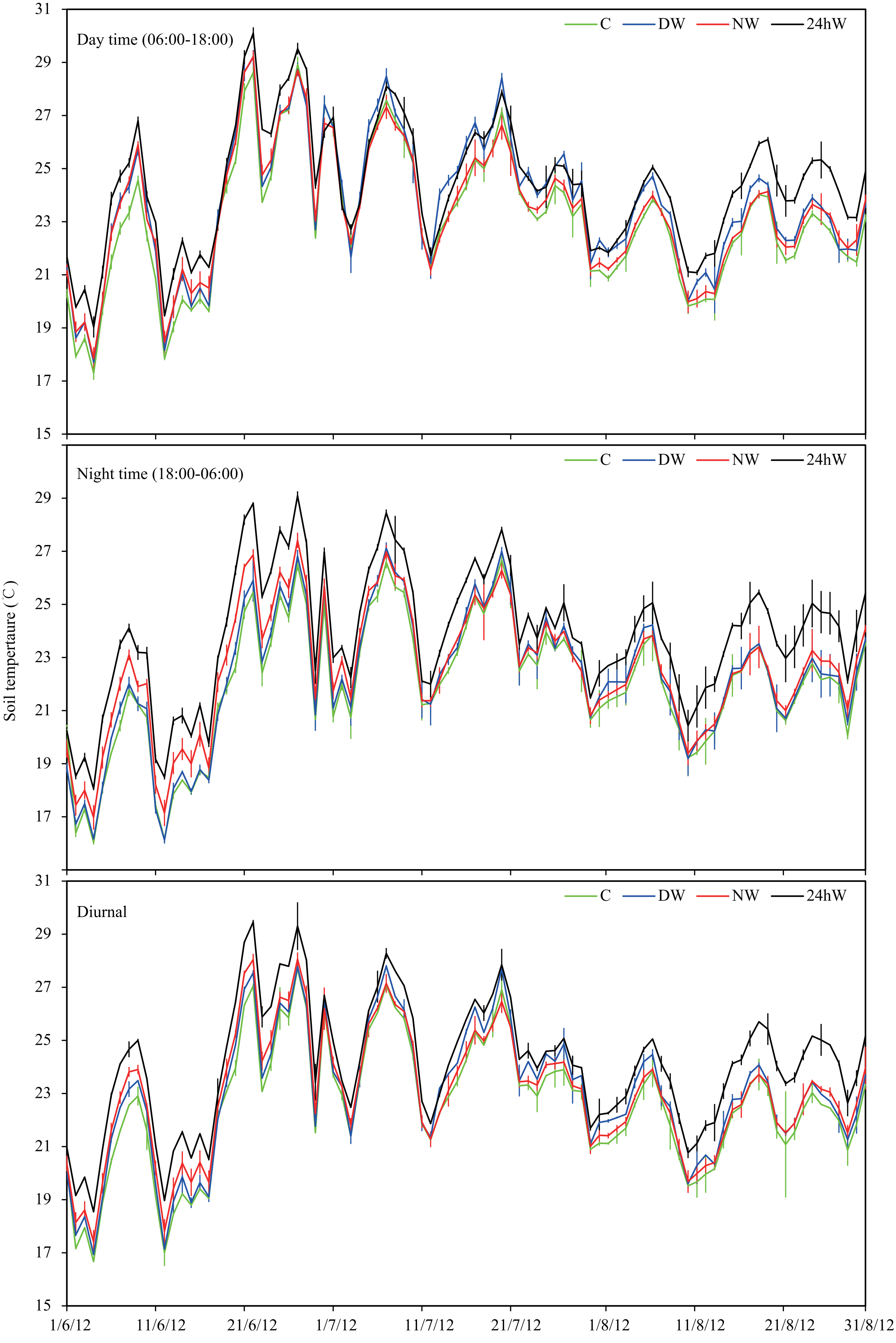

Day time (06:00-18:00)
Night time (18:00-06:00)
Diurnal
C
DW
NW
24hW
Soil tempertaure (°C)
31
29
27
25
23
21
19
17
15
1/6/12
11/6/12
21/6/12
1/7/12
11/7/12
21/7/12
1/8/12
11/8/12
21/8/12
31/8/12

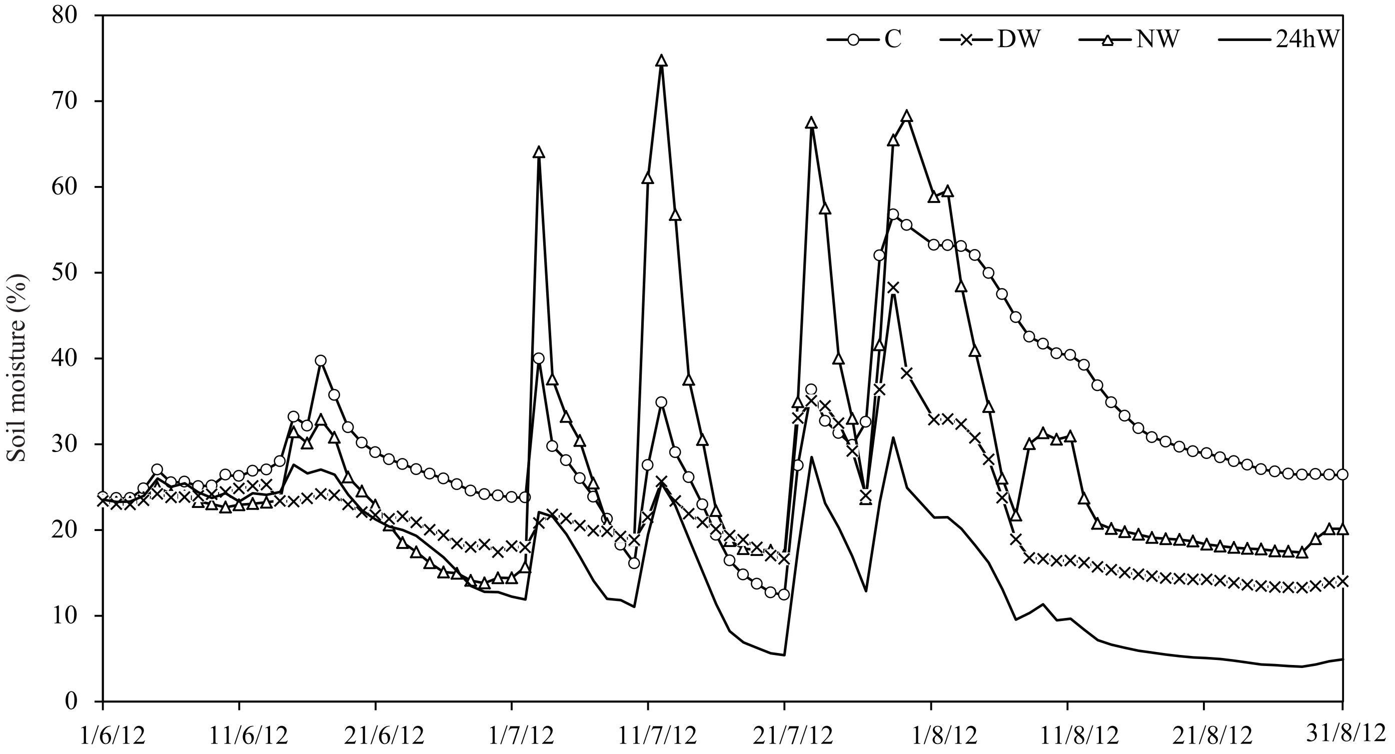
Soil moisture (%)
80
70
60
50
40
30
20
10
0
C
DW
NW
24hW
1/6/12
11/6/12
21/6/12
1/7/12
11/7/12
21/7/12
1/8/12
11/8/12
21/8/12
31/8/12
